# Unraveling the mystery of ocular retinoid turnover: Insights from albino mice and the role of STRA6

**DOI:** 10.1016/j.jbc.2024.105781

**Published:** 2024-02-21

**Authors:** Srinivasagan Ramkumar, Beata Jastrzebska, Diego Montenegro, Janet R. Sparrow, Johannes von Lintig

**Affiliations:** 1 Department of Pharmacology, School of Medicine, Case Western Reserve University, Cleveland, Ohio, USA; 2 Department of Ophthalmology, Columbia University Medical Center, New York, New York, USA; 3 Departments of Pathology and Cell Biology, Columbia University Medical Center, New York, New York, USA

**Keywords:** vision, photoreceptors, retinal pigment epithelium, retinoids, metabolism

## Abstract

A delicate balance between photon absorption for vision and the protection of photoreceptors from light damage is pivotal for ocular health. This equilibrium is governed by the light-absorbing 11-*cis*-retinylidene chromophore of visual pigments, which, upon bleaching, transforms into all-*trans*-retinal and undergoes regeneration through an enzymatic pathway, named the visual cycle. Chemical side reactions of retinaldehyde during the recycling process can generate by-products that may result in a depletion of retinoids. In our study, we have clarified the crucial roles played by melanin pigmentation and the retinoid transporter STRA6 in preventing this loss and preserving the integrity of the visual cycle. Our experiments initially confirmed that consecutive green and blue light bleaching of isolated bovine rhodopsin produced 9-*cis* and 13-*cis* retinal. The same unusual retinoids were found in the retinas of mice exposed to intense light, with elevated concentrations observed in albino mice. Examining the metabolic fate of these visual cycle byproducts revealed that 9-*cis*-retinal, but not 13-*cis*-retinal, was recycled back to all-*trans*-retinal through an intermediate called isorhodopsin. However, investigations in *Stra6* knockout mice unveiled that the generation of these visual cycle byproducts correlated with a light-induced loss of ocular retinoids and visual impairment. Collectively, our findings uncover important novel aspects of visual cycle dynamics, with implications for ocular health and photoreceptor integrity.

Photoreceptors, specialized neurons located within the retina, play a critical role in our visual perception. These remarkable cells are responsible for detecting light and translating it into nerve signals (1). The unique light-absorbing properties of photoreceptors hinge on the presence of visual pigments, which are harbored within specialized membranous compartments known as the outer segments of these cells (2). These visual pigments are intricate molecules, essentially made up of two key components: an opsin protein moiety and a covalently bound 11-*cis*-retinylidene chromophore (3).

When a photon strikes visual pigments, geometric isomerization occurs within the chromophore, resulting in the creation of metarhodopsin II. Metarhodopsin II, in turn, initiates a complex cascade of events known as phototransduction, involving a protein called transducin (1). To return visual pigments to their ground state, metarhodopsin eventually decays into opsin and all-*trans*-retinal. The regeneration of chromophores happens through a multi-step pathway named the visual cycle, as originally described by Wald (4). This process unfolds within the outer segments of photoreceptors and the adjacent retina pigment epithelium (RPE), relying on various enzymes such as retinal dehydrogenases, lecithin retinol acyltransferase, and the retinoid isomerase RPE65 (5, 6, 7). Furthermore, retinoid binding and transport proteins facilitate the transportation of the visual cycle intermediates between the photoreceptor outer segments and the RPE (8).

Given the high metabolic flux of retinoids within the visual cycle and the oxygen- and light-rich environment of photoreceptors, it is not surprising that ocular retinoids are susceptible to chemical side reactions. For instance, light stress induces the generation of visual cycle by-products, such as 9-*cis* and 13-*cis*-retinal, in the mouse retina (9). Intriguingly, these by-products persist in the retina for extended periods post-bleaching, although their precise metabolic fate remains unresolved. Notably, retinaldehydes exhibit photosensitizing properties and generate free radicals capable of causing harm to cellular components (10). Moreover, the carbonyl group of retinaldehyde can engage in reactions with phosphatidylethanolamine, leading to the formation of bisretinoid adducts, notably the pyridinium bisretinoid A2E (11). A2E, recognized for imparting golden-yellow fluorescence to lipofuscins in the eyes, is considered phototoxic to both the retina and the retinal pigment epithelium (RPE) (Sparrow *et al.*, 2020). However, it remains inconclusive whether these chemical side reactions of retinaldehydes contribute measurably to the loss of ocular retinoids.

We performed biochemical and mouse studies to unravel the underlying processes. Initially, we demonstrated with isolated bovine rhodospin that exposure to the green and blue light generated a novel product with a peak absorption at 485 nm. This transformation coincided with the generation of 9-*cis*, 11-*cis*, and 13-*cis*-retinal. Extending our inquiry to an animal model, we revealed similar photochemical reactions occurring within the mouse retina upon exposure to intense white light. Notably, the formation of 9- and 13-*cis*-retinaldehyde was more pronounced in albino mice, lacking melanin pigmentation. In the case of albino mice, our observations indicated a correlation between the formation of these compounds and a consequential loss of retinoids within the eyes. Through a comparative analysis involving wild-type (WT) and mutant mice, our study revealed a crucial role of the retinoid transporter encoded by the Stimulated retinoic acid six gene (Stra6). This transporter emerges as indispensable in compensating for the light-dependent loss of ocular retinoids by facilitating the reuptake of vitamin A from the circulation.

## Results

### Photochemical reactions of isolated bovine rhodopsin

**Figure 1:**
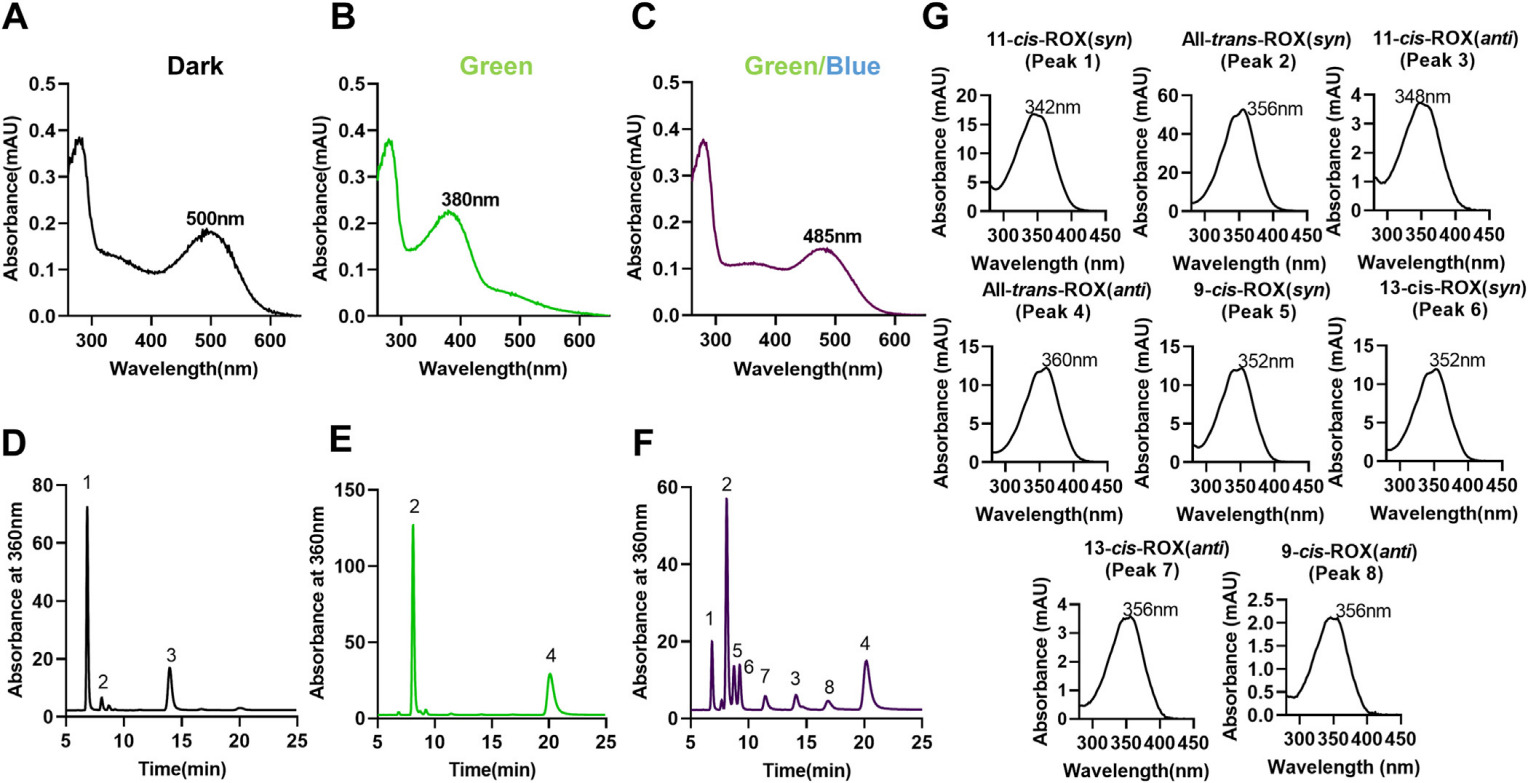
**Green and blue light bleaching of bovine rhodopsin.** UV-visible spectra of isolated bovine rhodopsin preparation. *A*, dark-adapted, (*B*) monochromatic green (λ = 535 nm) light exposed, (*C*) monochromatic green and blue (λ = 405 nm) light exposed. *D*–*F*, HPLC trace at 360 nm of retinoid extracts isolated from of dark–adapted (*D*), monochromatic green (*E*), and monochromatic *green* and *blue light* (*F*) exposed bovine rhodopsin preparations, respectively. The absorbance maxima of each spectrum were pointed. The retinal diastereomers were converted to corresponding retinal oximes (ROX) during extraction and the corresponding oximes exist as *syn* and *anti*-isomers. The individual retinal peaks (*D*–*F*) were numbered and the corresponding spectra of the different retinaloxime diastereomers (*syn* and *anti*) are shown in *panel G*.

We previously, reported that successive bleaching with green and blue laser light generates 9-*cis* and 13-*cis*-retinal in mouse eyes (9). We now analyzed the biochemistry of this isomerization reaction. For this purpose, we isolated native rhodopsin from the rod outer segments of bovine retinas using lipid solubilization with β-D-maltopyranoside, following the procedure described in a previous study (12). In the spectroscopic examination of the dark-adapted rhodopsin preparation, a distinctive spectrum with an absorption peak at 500 nm was observed (Fig. 1*A*). When we subjected rhodopsin to saturating illumination with monochromatic green light (535 nm) at 4 °C, the absorption spectrum underwent a notable shift to a new peak at 380 nm (Fig. 1*B*). This shift indicated the conversion of rhodopsin into metarhodopsin II. We then repeated the green light bleaching of the dark-adapted rhodopsin, but this time we included an additional 10-s exposure to monochromatic blue light (405 nm). The spectroscopic analysis of the green-blue bleached rhodopsin revealed a change in the absorption maximum, characterized by a bathochromic shift leading to a novel absorption peak at 485 nm (Fig. 1*C*).

To gain insights into the underlying retinoid chemistry, we subjected the rhodopsin preparations exposed to different illumination conditions to retinoid extraction using organic solvents. During this extraction process, retinaldehyde diastereomers underwent conversion into the respective oximes through the action of hydroxylamine. Consequently, the HPLC traces revealed the presence of retinaldehyde diastereomers in both *syn*- and *anti*-configurations.

In the rhodopsin preparations adapted to darkness, the major retinaldehyde diastereomer observed was 11-*cis*-retinal, with a small trace of all-*trans*-retinal becoming detectable in the isolated rhodopsin preparation (Fig. 1*D*). Following exposure to green light, the 11-*cis*-retinal was nearly completely transformed into all-*trans*-retinal, which serves as an indicator of metarhodopsin II formation (Fig. 1*E*). Analysis of preparations subjected to green/blue light revealed a mixture of various retinaldehyde diastereomers (Fig. 1*F*). The corresponding spectra for the retinaloxime peaks are presented in Figure 1*G*. Notably, we discovered, in addition to all-*trans*-retinal, significant quantities of 9-*cis*, 11-*cis*, and 13-*cis*-retinal.

To investigate whether the observed light-dependent *trans*/*cis* isomerization of all-*trans*-retinal occurred in its bound or unbound state, we introduced hydroxylamine into our rhodopsin preparations and assessed their absorption spectrum. Hydroxylamine has the ability to react with unbound retinaldehyde to produce the corresponding retinaloximes (measuring at 360 nm). The introduction of hydroxylamine did not result in any changes in the absorption spectrum of dark-adapted rhodopsin (Fig. S1*A*). Following green light exposure, rhodopsin was transformed into metarhodopsin II. When hydroxylamine was present, metarhodopsin II exhibited partial decay into opsin and retinaldehyde, which were then converted into the corresponding oxime (360 nm) (Fig. S1*B*). During the green/blue light exposure of metarhodopsin II in the presence of hydroxylamine, there was a slight reduction in the quantity of the newly formed rhodopsin species with an absorption peak at 485 nm when compared to the sample without hydroxylamine treatment. Consequently, a new peak with a maximum at 360 nm, characteristic of retinaloxime, emerged (Fig. S1*C*). These experimental results suggest that all-*trans*-retinal existed in both its opsin-bound and opsin-unbound forms following green light illumination (Fig. S1*C*), with the opsin-bound form being transformed into the new rhodopsin species exhibiting an absorption peak at 485 nm upon exposure to blue light.

### Formation of 9-*cis* and 13-*cis*-retinal in the mouse retina

We were next curious whether similar light-dependent isomerization reactions occur in native mouse photoreceptors under bright light illumination. To explore this, our focus was on the generation of 9-*cis* and 13-*cis*-retinal diastereomers, as they can be differentiated from the conventional retinoid cycle intermediates, such as all-*trans* and 11-*cis*-retinal, in HPLC analysis. In these experiments, we utilized albino and pigmented C57BL/6J mice, considering that ocular melanin content has an impact on phototransduction gain and recovery, as established by previous studies (13, 14).

Consequently, we exposed dark-adapted mice to 1 min of intense white light. Subsequently, the mice were placed in darkness and then sacrificed. We extracted ocular retinoids from their eyes for separation *via* HPLC. The HPLC chromatograms of the retinoid extract from dark-adapted albino and pigmented mice predominantly exhibited retinyl esters (REs) and 11-*cis*-retinal as the primary retinoids, with smaller quantities of all-*trans*-retinal and all-*trans*-retinol (as depicted in Fig. S2, *A* and *B*).

In the mice exposed to light, in addition to the established visual cycle intermediates REs, 11-*cis*, and all-*trans-*retinal, notable amounts of 9-*cis* and 13-*cis-*retinal were detected (as shown in Fig. S2*B*). It is worth noting that the concentration of 9-*cis* and 13-*cis*-retinal was significantly higher in the retinas of albino mice when compared to pigmented mice (as presented in Fig. S2, *C* and *D*). This implies that non-standard retinoid cycle intermediates were generated in mouse retinas following exposure to intense white light, and their concentration was elevated in albino mouse retinas.

### In the mouse retina, 9-*cis*-retinal exists in opsin bound form

We proceeded to investigate the metabolic destiny of 13-*cis* and 9-*cis*-retinal. Specifically, we explored how 9-*cis*-retinal behaves when it interacts with opsin, forming isorhodopsin (with a maximum absorption at 485 nm) that can be transformed into metarhodopsin II upon exposure to longer wavelength light (15). Notably, the spectral characteristics of opsin-bound 9-*cis*-retinal (isorhodopsin) can be distinguished from those of opsin-unbound 9-*cis*-retinal (maximum absorption at 362 nm). We harnessed this disparity to determine the form in which 9-*cis*-retinal existed in the mouse retina.

Our experimental approach involved initially exposing dark-adapted albino mice to intense white light for 1 min to generate 9-*cis*-retinal. After a subsequent five-minute dark adaptation period, we exposed the mice to monochromatic green light (535 nm). This particular light selectively isomerizes isorhodopsin (maximum absorption at 485 nm) to metarhodopsin II while leaving free 9-*cis*-retinal (maximum absorption at 362 nm) unaffected.

**Figure 2:**
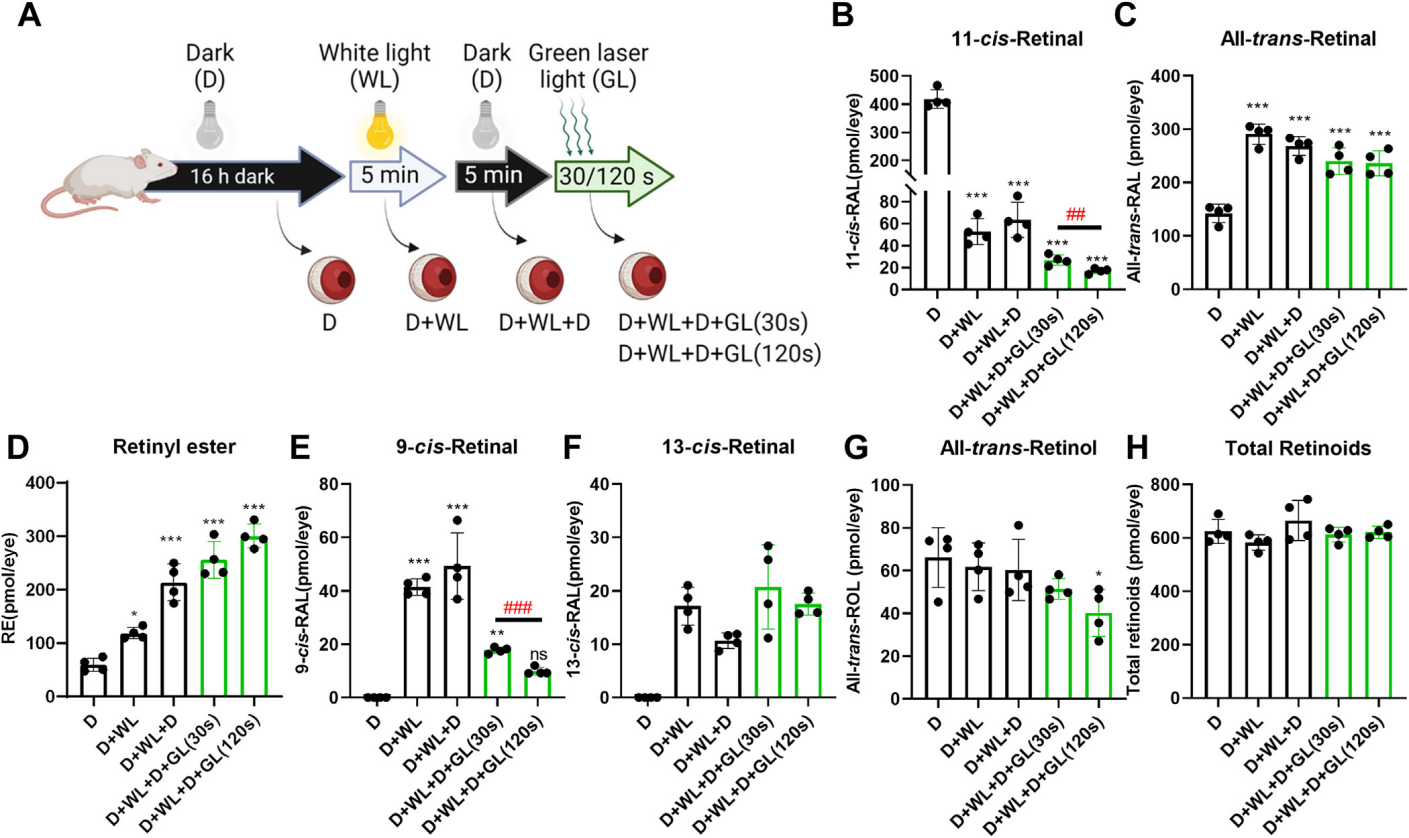
**Evidence for isorhodopsin generation in the mouse retina.***A*, scheme of the bleaching experiment. *B*–*H*, concentrations of the different retinoids extracted from the dissected eyes of albino WT mice subjected to different light conditions. *D*, dark-adapted for 16 h; D+WL, dark-adapted for 16 h and 1 min bright white light; D+WL+D, Dark-adapted for 16 h; 1 min bright white light, and 5 min dark-readaption; D+WL+D+GL, dark-adapted for 16 h, 1 min bright white light, 5 min dark-readaption, and monochromatic green light (λ = 532 nm) for 30 and 120 s (*B*–*H*) The retinoid cycle intermediates between groups were compared and values are displayed as mean ± SD. Statistical analyses (*n* = 4) were performed by comparing values of *D* to the different light treatment conditions (∗*p* < 0.05, ∗∗*p* < 0.005, ∗∗∗*p* < 0.0001) using one-way ANOVA by comparing the mean of each column with the mean of a dark-adapted column and Dunnett test as post hoc analysis and by comparing the values between D + W + D+GL 30 and 120 s (##*p* < 0.005, ###*p* < 0.0001) using unpaired two-tailed Student’s *t* test. RAL, retinaldehyde; RE, retinyl esters; ROL, retinol.

Analysis of ocular retinoids using HPLC for different treatment groups (Fig. 2*A*) revealed that bright white light bleaching led to a decrease in 11-*cis*-retinal and a corresponding increase in all-*trans*-retinal (Fig. 2, *B* and *C*). Furthermore, approximately 40 and 20 pmol per eye of 9-*cis*- and 13-*cis*-retinal were generated following the bright white light bleach (Fig. 2, *D* and *E*). After a five-minute dark adaptation, the levels of 9-*cis*- and 13-*cis*-retinal diastereomers remained constant (Fig. 2, *D* and *E*). In contrast, the concentration of all-*trans*-retinal decreased while the concentration of retinyl esters (RE) increased (Fig. 2, *C* and *F*), indicating that the recycling of the canonical bleaching product, all-*trans*-retinal, proceeded in the eyes of dark-readapted mice.

In mice subjected to additional green light bleaching (30 s and 120 s), the concentration of 9-*cis*-retinal progressively decreased to approximately 18 and 10 pmol per eye after exposure to the green light bleach (Fig. 2*D*, GL), signifying its existence as isorhodopsin. Moreover, the remaining 11-*cis*-retinal concentration significantly diminished, indicating photobleaching of the remaining rhodopsin by the monochromatic green light (Fig. 2*B*, GL). In contrast, the concentration of 13-*cis*-retinal remained unaffected following the green light bleach (Fig. 2*E*). The total retinoid concentration remained constant across the treatment groups (Fig. 2*H*), suggesting that the variations in retinoid diastereomer composition were not due to random effects.

To ensure that the monochromatic green light source did not isomerize opsin-unbound retinaldehyde, we conducted an additional control experiment. We illuminated a 9-*cis*-retinal solution (10 μM) with both green (λ = 535 nm) and ultraviolet (λ = 405 nm) light (Fig. S3*A*). After illumination, we employed HPLC to determine the geometric composition of the retinaldehyde diastereomers. Importantly, green laser light had no impact on the geometric composition of 9-*cis*-retinal (Fig. S3*B*). In contrast, deep blue laser light isomerized 9-*cis*-retinal into a mixture of 11-*cis*, 13-*cis*, and all-*trans*-retinal (Fig. S3*B*). Therefore, we confirmed the integrity of the green laser light source in our experiments and concluded that 9-*cis*-retinal was bound at least in part to opsins in the mouse retina upon the initial white light illumination.

### Melanin pigmentation affects ocular retinoid concentrations in STRA6-deficient mice

Our investigations unveiled an enhanced generation of 9-*cis* and 13-*cis*-retinal diastereomers in the retina of albino mice. While 9-*cis*-retinal can be at least in part regenerated *via* isorhodospin, 13-*cis*-retinal might be excluded from the recycling process and undergo reactions with other molecules (9). This led us to ponder whether there is a light-dependent depletion of retinoids in the eyes of albino mice caused by such reactions. To address this question, we capitalized on the use of *Stra6*^*−/−*^ mice, which have impaired ocular vitamin A uptake homeostasis (16, 17). We postulated that a light-dependent loss of retinoids would become measurable in albino STRA6-deficient mice due to a compromised reuptake of the retinol from circulating retinol binding protein (RBP4).

**Figure 3:**
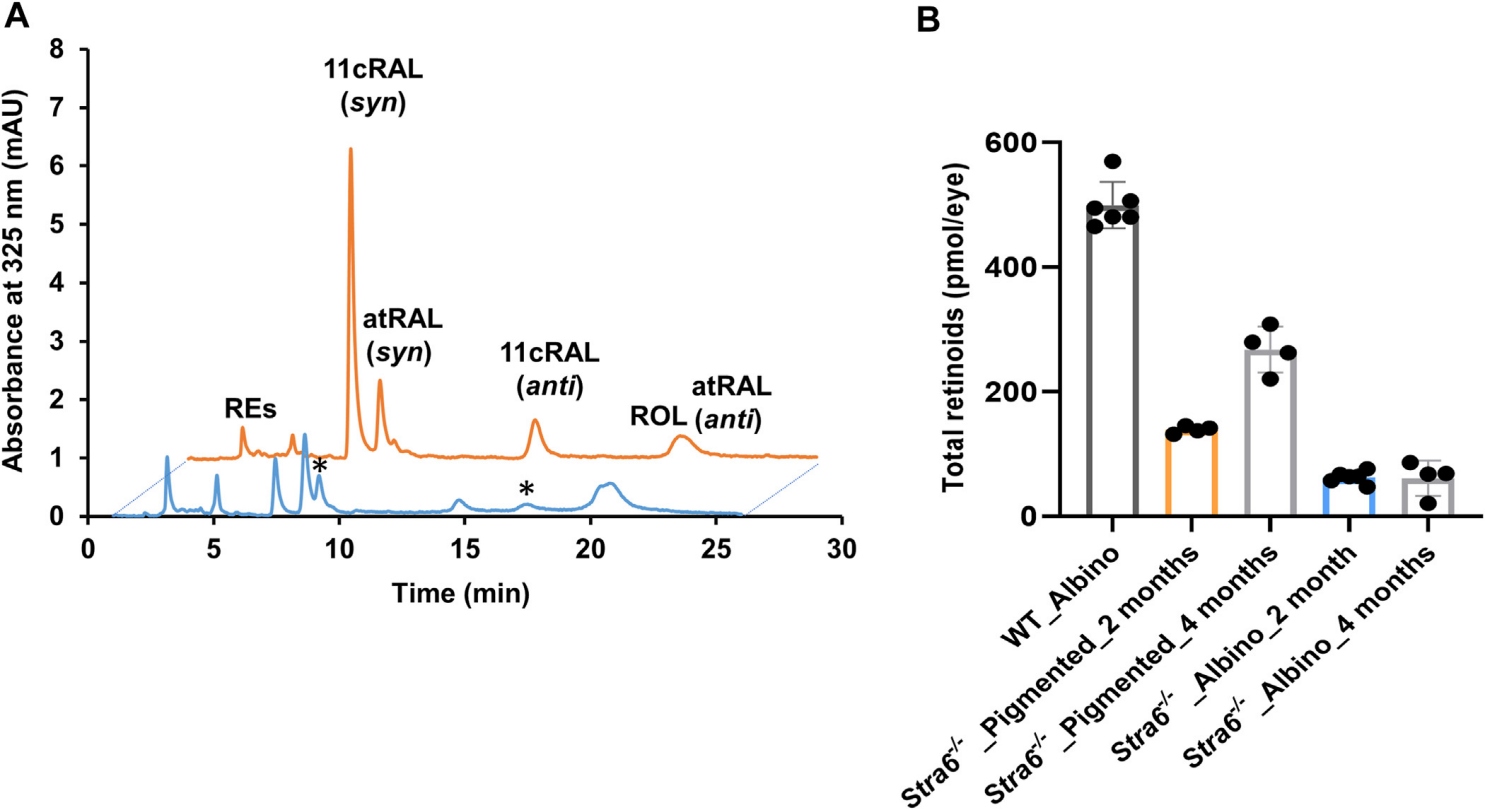
**Ocular retinoid content of *Stra6* knockout is influenced by melanin pigmentation.***A*, HPLC traces at 325 nm of 2-months-old, dark-adapted albino *Stra6*^−/−^ (*blue trace*) and pigmented *Stra6*^*−/−*^ mice (*orange trace*). Retinaldehydes were converted to the corresponding retinaloximes (ROX) during the extraction and exist as *syn* and *anti*-isomers. The asterisks indicate peaks for 9-*cis* and 13-*cis*-retinal oxime (syn and anti) in HPLC traces of albino *Stra6*^*−/−*^ mice. *B*, total ocular retinoid concentration of 2 months old albino WT and 2- and 4-months old pigmented and albino *Stra6*^−/−^ mice (*n* = 4–6 each). Values are displayed as mean ± SD ∗∗∗*p* < 0.0001. The statistical analysis was performed by unpaired two-tailed Student’s *t* test. 11cRAL, 11-*cis*-retinal-oxime; atRAL, all-trans-retinal oxime; REs, retinyl esters; RO, all-*trans*-retinol.

To investigate this hypothesis, we crossed the *Stra6* gene into the genetic background of B6(Cg)-Tyrc-2 J/J mice, establishing an albino STRA6-deficient mouse line. In initial experiments, we assessed the ocular retinoid concentration in mice raised on a 12/12 light-dark cycle for 2 months using HPLC analysis. Notably, the ocular retinoid concentration in albino *Stra6*^*−/−*^ mice was only one-third of that found in age-matched pigmented *Stra6*^*−/−*^ mice (Fig. 3, *A* and *B*). Remarkably, ocular retinoid concentrations increased in 4-months old pigmented *Stra6*^*−/−*^ mice while ocular retinoid concentrations remained at a very low level in adolescent albino *Stra6*^*−/−*^ mice (Fig. 3*B*). As expected, no such differences were observed in albino and pigmented WT mice (Fig. 3, *A* and *B*).

All retinoid cycle intermediates were detected in albino *Stra6*^*−/−*^ mice, although, similar to pigmented *Stra6*^*−/−*^ mice, they displayed very low levels of RE. Additionally, the HPLC traces of albino *Stra6*^*−/−*^ mice displayed peaks for 9-*cis* and 13-*cis*-retinal, whereas these retinaldehyde diastereomers were barely detectable in pigmented *Stra6*^*−/−*^ mice (Fig. 3*A*, see asterisks). Therefore, we verified our hypothesis that pigmentation had a significant effect on ocular retinoid concentration and composition in STRA6-deficient mice.

### Loss of ocular retinoids in albino *Stra6*^*−/−*^ mice increases the concentration of unliganded opsin

**Figure 4:**
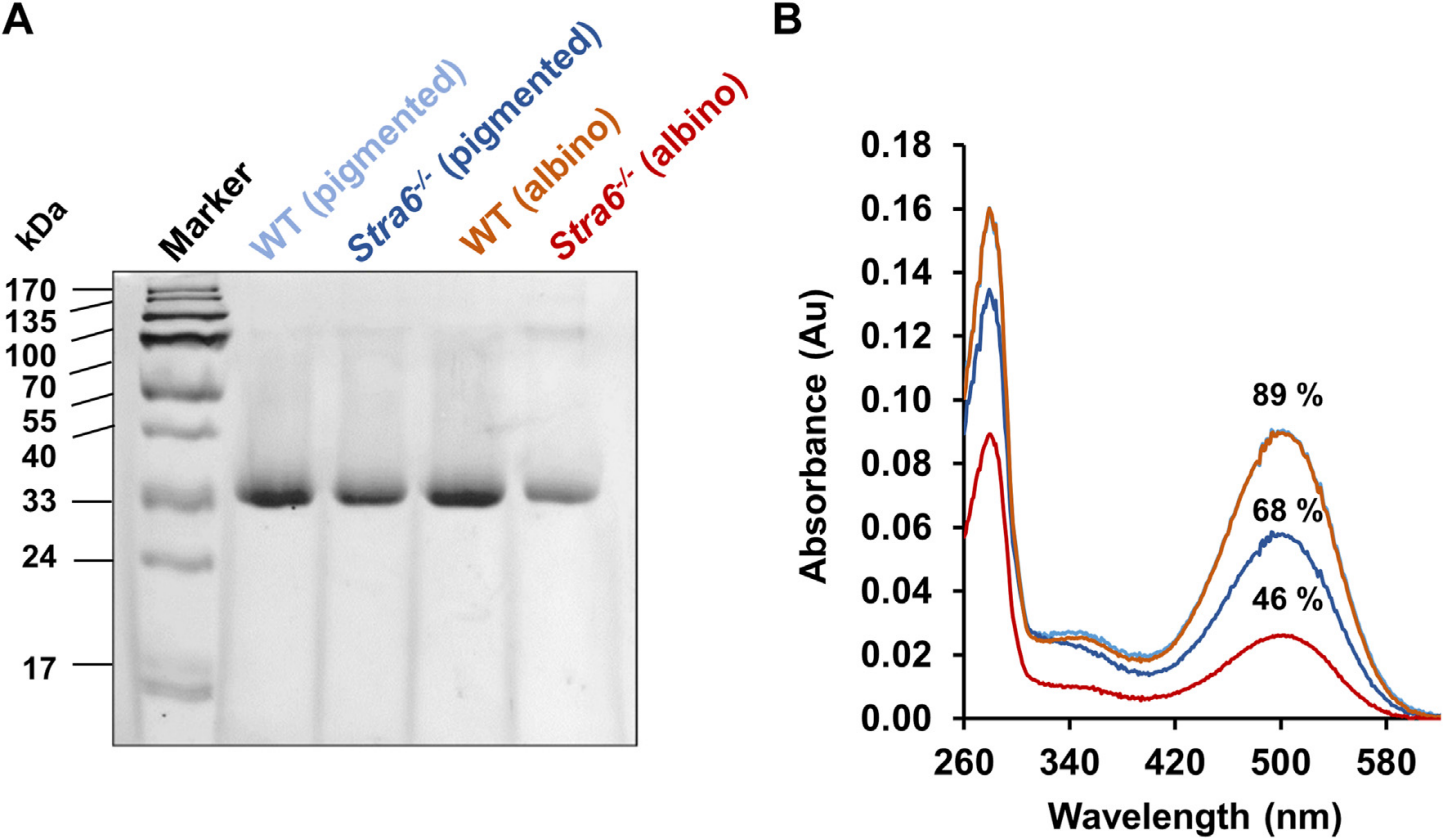
**Albino STRA6-deficient mice exhibit higher concentration of unliganded rhodopsin.***A*, 12% SDS-PAGE of the 1D4 immune-purified rhodopsin fraction of 2-months old albino and pigmented WT and Stra6 ^−/−^ mice. *B*, UV-visible spectra of the immune-purified rhodopsin fractions for 2-month-old albino and pigmented WT and Stra6 ^−/−^ mice. Please note that the rhodopsin spectra from pigmented and albino mice overlap. The percentage in *panel B* percentage pertains to the 280/500 nm ratio of the isolated rhodopsin from the different *mouse lines*.

We previously reported that the photoreceptors in pigmented *Stra6*^*−/−*^ mice exhibit unbound opsin and a diminished sensitivity to light (18, 19, 20). This particular trait is associated with a constitutive activation of sensory transduction, ultimately leading to the demise of photoreceptor cells (21). Consequently, we next postulated that the reduced retinoid concentrations of albino *Stra6*^*−/−*^ mice exacerbate the phenotype. Therefore, we examined immuno-purified rhodopsin from various mouse lines utilizing SDS-PAGE. The analysis revealed that the opsin content in the retinas of two-months-old STRA6-deficient albino and pigmented mice, raised in well-lit conditions, was notably lower compared to age-matched albino and pigmented WT mice (Fig. 4*A*). This reduction was mirrored in a significant lower expression of *Rho* gene in both albino and pigmented *Stra6*^*−/−*^ mice as compared to the respective WT controls (Fig. S4*A*). Similarly, levels of *Opn1mw* and *Opn1sw*, encoding M- and S-cone opsins were lower in *Stra6*^*−/−*^ mice (Fig. S4, *B* and *C*). The expression of the genes encoding G protein subunit alpha transducin 1 (Gnat1) and transducin 2 (Gnat2) followed the same patterns (Fig. S4, *D* and *E*). Thus, *Stra6*^*−/−*^ mice displayed reduced expression of the rod and cone phototransduction machineries but pigmentation had no influence on their mRNA levels.

We next conducted UV-visible spectrophotometry on isolated protein, comparing the theoretical 280/500 nm ratio of rhodopsin with the actual absorption ratio of the individual samples as previously described (22). The results indicated that 89% of opsin existed in its chromophore-bound form in both 2-month-old dark-adapted pigmented and albino WT mice. Pigmented *Stra6*^*−/−*^ mice exhibited a substantial decrease in the chromophore-bound opsin fraction, down to 64% (see Fig. 4*B*). In the case of albino *Stra6*^*−/−*^ mice, the 280 to 500 nm ratio was further skewed toward free opsin when compared to WT mice and only 45.5% of opsin existed in chromophore-bound form (Fig. 4*B*). Consequently, albino *Stra6*^*−/−*^ mice, displayed a further decrease of ligand-bound rhodopsin to 46%, a trend that correlated with their low ocular retinoid concentrations (Fig. 3*B*).

We also recorded the electrical responses in dark-adapted eyes and the responses in light-adapted eyes by electroretinography (ERG) (23, 24) (Fig. S5). In *Stra6*^*−/−*^ mice, scotopic ERG responses (a- and b-waves) to flash light stimuli were highly diminished in comparison to WT mice, with a trend to be further decreased in albino *Stra6*^*−/−*^ mice (Fig. S5, *A* and *B*). As previously reported (18), under photopic conditions, ERG responses were barely detectable even at the highest illuminance in albino and pigmented *Stra6*^*−/−*^ mice when compared to WT control mice (Fig. S5).

### The loss of ocular retinoids in albino *Stra6*^*−/−*^ mice is light-dependent

**Figure 5:**
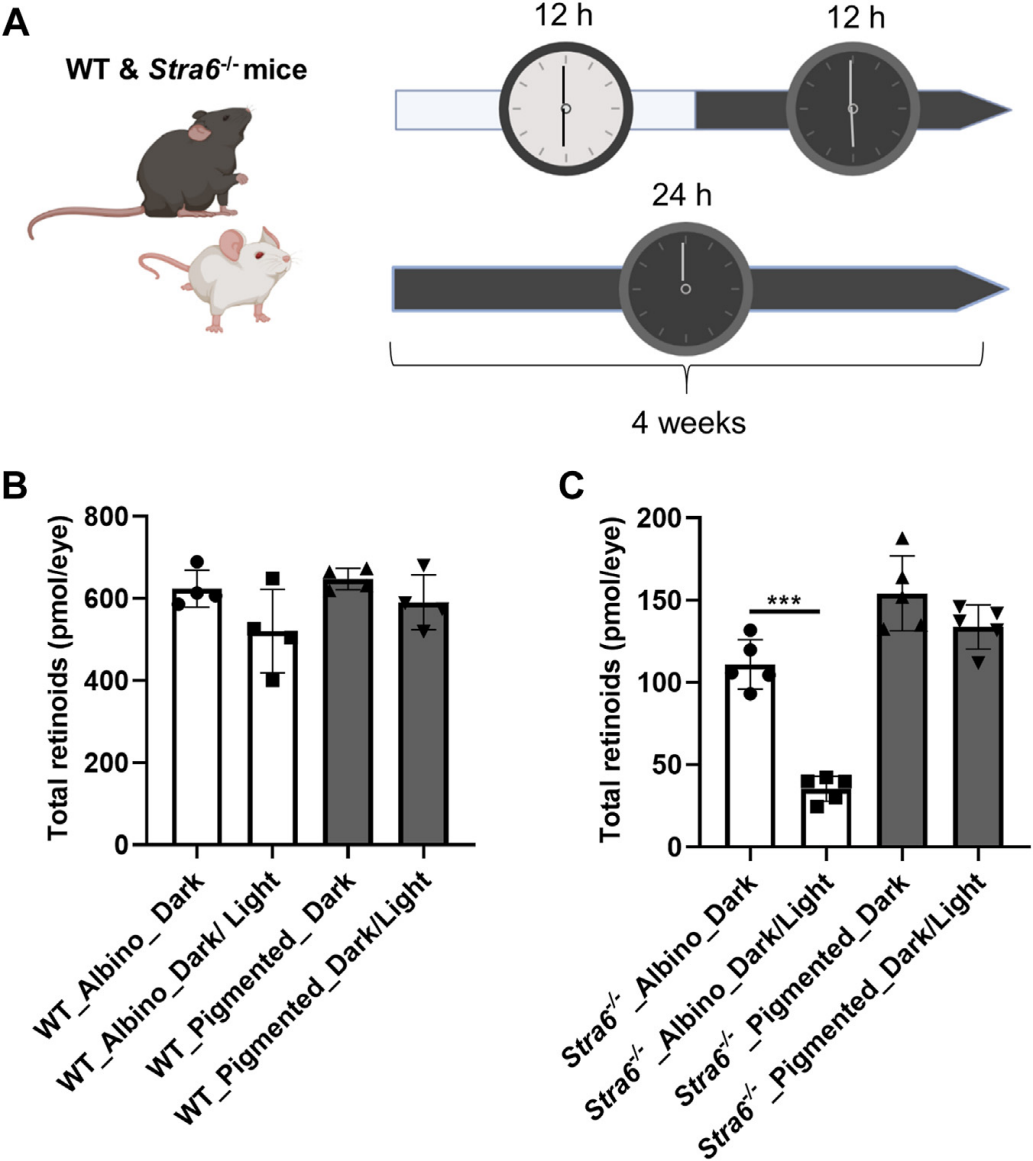
**Light-dependent decrease of ocular retinoid concentration of albino STRA6-deficient mice.***A*, scheme of the experiment. *BandC*, total ocular retinoid concentration of pigmented and albino WT and *Stra6*^−/−^ mice (*n* = 4–5 each) raised in a light dark cycle (16/8 h) or in constant darkness. The values are displayed as mean ± SD ∗∗∗*p* < 0.0001. The statistical analysis was performed by unpaired two-tailed Student’s *t* test.

To explore whether light exposure is causative for the low ocular concentration of albino *Stra6*^*−/−*^ mice, we placed freshly weaned pigmented and albino *Stra6*^*−/−*^ mice on a 12/12 light-dark cycle or in complete darkness. Additionally, we included albino and pigmented WT mice as control subjects for this experiment. After a 4-week period, we euthanized the animals and quantified ocular retinoid concentrations using HPLC analysis (Fig. 5*A*).

The results indicated that WT mice maintained consistent ocular retinoid levels regardless of their pigmentation or the ambient light conditions they were exposed to (Fig. 5*B*). In contrast, *Stra6*^*−/−*^ mice displayed significantly lower ocular retinoid concentrations compared to WT mice (Fig. 5, *B* and *C*). Particularly, the ocular retinoid concentration in albino *Stra6*^*−/−*^ mice exposed to light was more than three times lower than that of their counterparts raised in darkness (Fig. 5*C*). There also was a slight variation between pigmented *Stra6*^*−/−*^ mice raised in dark and light conditions, although this difference did not reach statistical significance in the analysis (Fig. 5*C*). Consequently, we concluded that ambient light had a profound impact on the ocular retinoid concentration of albino *Stra6*^*−/−*^ mice but did not significantly affect albino WT mice. This discovery implies that the STRA6 transporter plays a crucial role in upholding a stable ocular concentration in albino mice in the presence of illumination.

### Light-dependent retinoid turnover is not associated with bisretinoid accumulation

We observed the light-dependent formation of unusual retinoids that was associated with retinoid depletion in albino STRA6-deficient mice. In the membranes of photoreceptors, retinaldehyde has the potential to form a Schiff base with phosphatidylethanolamine (PE), that upon reaction with a second retinaldehyde molecule leads to the production of bisretinoids (25). To investigate whether the retinoid decline in albino *Stra6*^*−/−*^ mice was linked to bisretinoid formation, we isolated these compounds from mice that were 3 months old and raised either in darkness or under a light-dark cycle. Subsequently, we analyzed the lipid extracts using a well-established UPLC protocol designed to detect bisretinoid lipofuscin pigments A2E and A2GPE, as well as the Schiff base N-retinylidene-PE (NRPE) (26).

**Figure 6:**
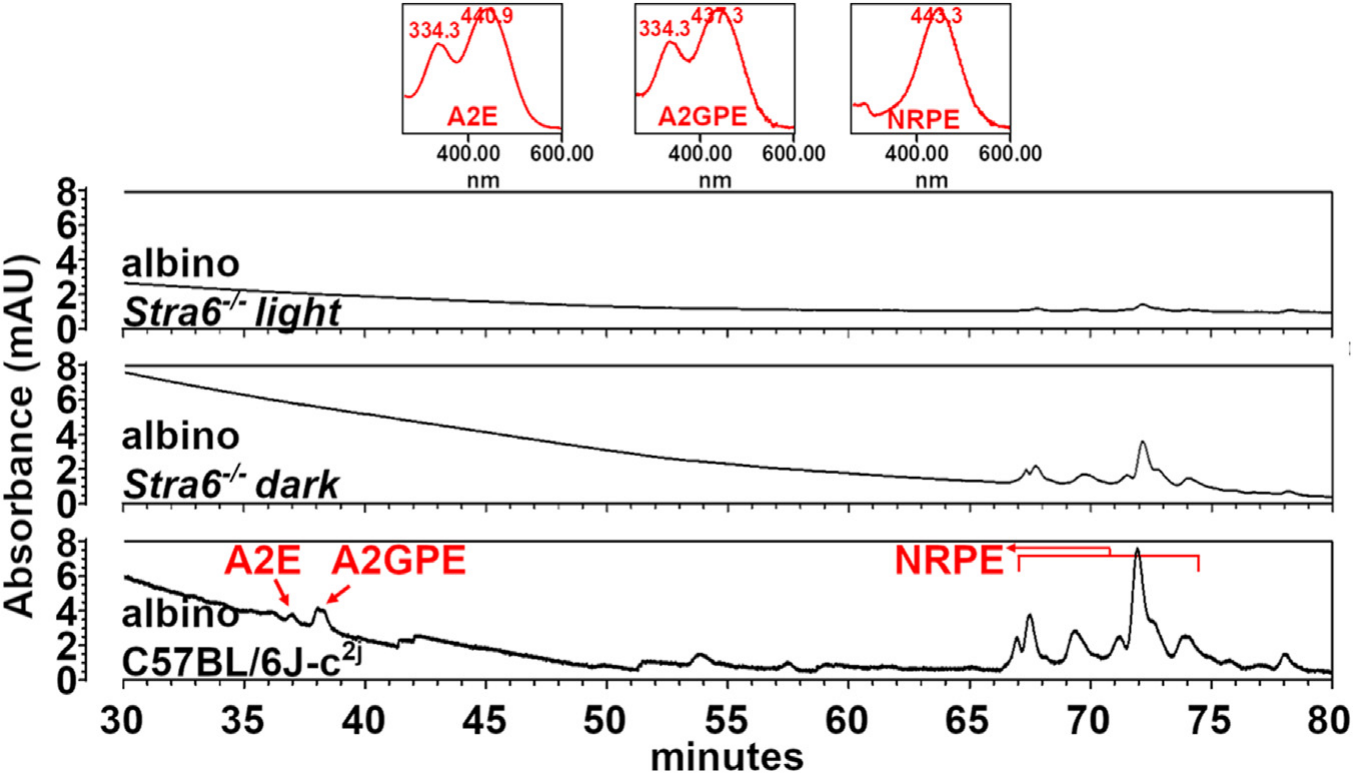
**Representative UPLC chromatograms illustrating the detection of the bisretinoid lipofuscin pigments A2E and A2GPE and the Schiff base N-retinylidene-PE (NRPE).** Samples consisted of albino *Stra6*^−/−^ cyclic light-reared (10 eyes), albino *Stra6*^−/−^ dark-reared (10 eyes), and albino C57BL/6J-c2j (4 eyes) control mice reared under cyclic light conditions. All mice were 3 months of age. Compounds were identified on the basis of UV-visible absorbance and retention times recorded using authentic standards. Insets (*top*), UV-visible absorbance spectra of chromatographic peaks corresponding to the bisretinoids A2E and A2GPE and N-retinylidene-PE (NRPE). A2E and A2GPE were not detected in the albino *Stra6*^*−/−*^ mice. Chromatograms were recorded by monitoring at 430 nm. Samples were prepared by homogenizing pooled eyes in a chloroform/methanol (1:1) mixture with subsequent centrifugation and filtering. Samples were then dried under argon and stored on −80 °C until UPLC analysis. mAU, milli-absorbance units.

Surprisingly, the hydrophobic extracts from the eyes of albino *Stra6*^*−/−*^ mice raised in the light did not contain any detectable amounts of bisretinoids, even when we combined the samples from 10 eyes for analysis. Only trace amounts of NRPE became detectable in the lipid extracts of the eyes of dark-raised albino *Stra6*^*−/−*^ mice. In contrast, albino WT mice displayed significant levels of various retinal conjugates in their eyes (Fig. 6). Thus, the bisretinoid content was found to be correlated with the total retinoid concentration in the eyes of the respective mouse lines. However, it was not enhanced in albino mice, presumably because of the photolytic cleavage of bisretinoids in the absence of melanin pigmentation (27).

## Discussion

Our study has unveiled the pivotal roles played by melanin pigmentation and the retinoid transporter STRA6 in maintaining ocular retinoid homeostasis and preserving the integrity of the visual cycle. We initiated our investigation by demonstrating that exposure to blue light induces the generation of 9-*cis* and 13-*cis*-retinaldehyde diastereomers in bovine rhodopsin preparations. Subsequently, we established that these atypical retinoids are also present in the retinas of mice following exposure to intense white light, with higher concentrations observed in the photoreceptors of albino mice. Remarkably, our analysis of albino mice revealed that the formation of these compounds is associated with a substantial loss of retinoids, leading to severe chromophore depletion in STRA6-deficient albino mice. In the following, we will discuss the findings in the context of the literature.

We observed with isolated bovine rhodopsin preparations that blue light converted metarhodopsin II into new rhodopsin species with an absorption maximum of 485 nm. Analysis of the retinoid chemistry of this process revealed that this conversion was associated with the production of 9-*cis*, 11-*cis*, and 13-*cis*-retinal (Fig. 1). Similar photochemical reactions of metarhodopsin II have been previously reported with isolated bovine rhodopsin (28). It was initially suggested that this reaction involves photoisomerization of the opsin-bound chromophore, a process termed “photoreversal” of bleaching (29). However, in-depth biochemical and structural studies have uncovered that vertebrate rhodopsin does not undergo the classical photoreversal reaction observed in invertebrate visual pigments (30). Instead, blue light with a wavelength of 420 nm converts metarhodopsin II (initially at 380 nm) into metarhodopsin III at 485 nm, featuring an all-*trans*-15-anti configuration of the opsin-bound chromophore (31, 32). Subsequently, light absorption converts metarhodopsin III back into metarhodopsin II or rhodopsin and isorhodopsin *via* thermally unstable 9/11-*cis*-15-syn intermediates (33). Hence, we propose that the new rhodopsin peak (485 nm) in our experiments is a mixture of several rhodopsin species.

Our study also revealed that the formation of 9-*cis* and 13-*cis*-retinal occurred in the mouse retina and that melanin pigmentation affected the generation of these compounds (Fig. S2). In accordance with the investigations with isolated bovine rhodopsin, 9-*cis*-retinal existed at least in part as isorhodopsin, as 9-*cis*-retinal concentrations in the mouse eyes decreased upon bleaching with monochromatic green light (Fig. 2) that can isomerize double bonds of the opsin-bound retinylidene chromophore but not of free 9-*cis*-retinal. A similar sequence of photochemical reactions likely provides an explanation for the blue light-dependent regeneration of visual pigments in the albino rat retina, as previously observed by Grimm and colleagues (34, 35).

While isorhodospin (9-*cis*-retinal) can be converted back into metarhodospin II (all-*trans*-retinal) by light, retinaldehyde diastereomers such as 13-*cis*-retinal might undergo other chemical reactions (36). For instance, retinaldehydes possess photosensitizing properties and can enter an excited triplet state, transferring excess energy to molecular oxygen to generate an excited state of oxygen, known as singlet oxygen (10). Some researchers suggest that this reaction generates significant amounts of singlet oxygen (36, 37). Moreover, retinaldehydes have been reported to generate free radicals upon illumination in the test tube (38). Additionally, the carbonyl group of retinaldehyde can react with primary amines of surrounding biomolecules in the photoreceptor outer segment and neighboring RPE cells (39). Several review articles describe the consequences of chemical reactions of retinaldehydes in photoreceptors and RPE (36, 40, 41). However, it has not been conclusively shown whether the reactions are associated with a depletion of ocular retinoids.

Our investigations utilizing albino STRA6-deficient mice have yielded primary evidence supporting a light-dependent depletion of retinoids. The *Stra6* gene, highly expressed in the retinal pigment epithelium (RPE), plays a pivotal role in maintaining homeostasis in ocular retinoid uptake (16, 17). Consequently, in the absence of STRA6, the eyes resort to a considerably slower and less specific delivery mechanism relying on post-meal retinyl esters within chylomicrons (42). In albino STRA6-deficient mice exposed to light during adolescence, we observed a markedly lower concentration of ocular retinoids at 2 and 4 months of age compared to their pigmented counterparts (Fig. 3). Notably, this phenotype was rescued when STRA6-deficient albino mice were raised in darkness (Fig. 4). These results indicate a substantial loss of ocular retinoids in the presence of illumination in the albino mouse retina. This deficiency becomes severe when there is an inability to replenish these retinoids through the reuptake of vitamin A from the bloodstream. These observations align with the analysis of radiolabeled retinoids in mice, which has revealed their continuous exchange between the eyes and the blood (43). This exchange provides a potential explanation for why mammals maintain constant holo-RBP4 levels in their circulations (44), as circulating retinol may serve as a constant source for STRA6-expressing cells to replenish their retinoids and maintain homeostasis. This homeostasis is crucial for maintaining the stoichiometry between opsins and chromophores in photoreceptors (18, 19). an equilibrium we observed to be severely disrupted in albino STRA6-deficient mice (Fig. 5).

In summary, our findings provide evidence of a measurable depletion of retinoids during the visual process in the absence of melanin pigmentation, necessitating replenishment of the retinoid pool by STRA6. Although we present evidence correlating the generation of 9-*cis* and 13-*cis*-retinal to this process, the specific form in which retinoids are expelled from the eyes remains uncertain. Possible pathways include diffusion, photochemical reactions, and the formation of adducts with lipids, nucleotides, and proteins. Interestingly, we did not observe elevated bisretinoid levels in the eyes of albino mice (Fig. 6). However, quantifying bisretinoid amounts in albino mice is challenging due to photolytic cleavage in the RPE in the absence of melanin pigmentation (27).

Clinical evidence suggests that iris color and melanin pigmentation influence the risk of developing chronic eye diseases such as age-related macular degeneration (45). Therefore, it is crucial to study the protective capabilities provided by melanin pigmentation in various eye regions, including the iris and RPE. Furthermore, the investigation of carotenoid pigments, integral to the photoreceptors in the human macula (46), has revealed their ability to mitigate the light-induced generation of 9-*cis* and 13-*cis*-retinaldehyde (9) as well as accumulation of bisretinoid in the mouse retina (47). Therefore, further research is essential to elucidate all aspects of light-dependent retinoid loss and its potential role in ocular disease states. Pigmented and albino STRA6-deficient mouse models will serve as valuable tools for these investigations.

## Experimental procedures

### Animals and housing

All mice experiments in these studies were conducted by protocols approved by Case Western Reserve University Institutional Animal Care and Use Committee. Male mice on a pigmented C57BL/6J and albino B6(Cg)-Tyrc-2J/J genetic background were used for this study. Wild-type (WT) mice were purchased from Jackson Laboratory. The generation of *Stra6*^−/−^ mice in the C57BL/6J background was previously described (16). The generation of *Stra6*^−/−^ mice on an albino B6(Cg)-Tyrc-2J/J background was carried out by conventional breeding between pigmented C57BL/6J *Stra6*^−/−^and albino B6(Cg)-Tyrc-2J/J WT mice. All mice were bred and raised on a standard chow diet consisting of 15,000 IU vitamin A/kg (Prolab RMH 3000, LabDiet) at a vivarium located at Case Western Reserve University. Mice were raised on a dark/light cycle to maintain circadian rhythm. The dark-raised mice were transferred immediately after weaning in a dark room for 4 weeks. At the end of the experimental period, mice were anesthetized using a drug cocktail of ketamine (20 mg/ml) and xylazine (1.7 mg/ml). Mice were sacrificed by cervical dislocation. The eyes were immediately dissected, snap frozen in liquid nitrogen, and stored at −80 °C until further analysis.

### HPLC analysis of ocular retinoids

Ocular retinoids were extracted from one entire eye as previously described (18). In brief, one whole eye was homogenized in 200 μl of 2 M hydroxylamine (pH 6.8), Retinoids were then extracted two times using a mixture of 200 μl methanol, 400 μl acetone, and 500 μl hexane. A normal-phase Zorbax Sil (5 μm, 4.6 × 150 mm) column was used for HPLC analysis. Chromatographic separation was achieved by isocratic flow of 10% ethyl acetate/90% hexanes (v/v). The HPLC was previously scaled with synthesized standard compounds to quantify the molar amounts of retinoids.

### *In vitro* 9-*cis*-retinal isomerization assay

For the *in vitro* isomerization study, 9-*cis*-RAL (Toronto Research Chemical, North York, ON, CA) was prepared in acetone (10 μM) in a glass cuvette. The cuvette containing 9-*cis-*RAL was exposed for 2 s to blue (405 nm) and green (532 nm) laser light with a narrow-band width and less than 5 mW output as previously described (9). The geometric composition of 9-*cis*-RAL isomers was determined using HPLC as described previously (9).

### Bovine rhodopsin illumination

Rhodopsin was extracted from the rod outer segments (ROS) isolated from bovine retinas with dodecyl-β-D-maltopyranoside (DDM) as described previously (12). Rhodopsin was diluted to a final concentration of about 0.2 mg/ml in a buffer composed of 20 mM bis-tris-propane, 120 mM NaCl, 0.5 mM DDM, pH 7.5. One ml of diluted rhodopsin was placed in the quartz cuvette, which was kept at 4 °C. The UV-visible spectrum of dark-adapted samples was measured before sample illumination. Then, the sample was illuminated with the green laser light for 5 min from a distance of 2 cm, followed by the UV-visible spectrum measurement. This light exposure resulted in the conversion of 11-*cis*-retinal-bound rhodopsin (λ max = 500 nm) to its all-*trans*-retinal-bound metarhodopsin II state (λ max = 380 nm). Next, the sample was illuminated with blue laser light for 2 s from a distance of 2 cm, followed by a measurement of the UV-visible spectrum. Illumination of metarhodopsin II resulted in the spectral change of the opsin state with an absorption maximum of 480 nm. The retinal composition of bovine rhodopsin was determined by HPLC.

### Bright light exposure of mice

2-month-old albino and pigmented WT mice (*n* = 5–6) were dark-adapted for 24 h for this experiment. Pupils were fully dilated with 1% tropicamide (Falcon Pharmaceuticals). Mice were anesthetized using a drug cocktail of ketamine (20 mg/ml) and xylazine (1.7 mg/ml) and mice eyes were exposed to the 85,000-lux 100 W LED white light (IP65, ZHMA) for 1 min. The dark-adapted mice not exposed to any light serve as control. Immediately after mice were sacrificed in the dark, eyes were enucleated. Ocular retinoid composition was determined by HPLC analysis (9).

### Laser light exposure of mice

2-month-old albino WT mice were used for this experiment. Mice were dark-adapted for 16 h, and then the pupils were fully dilated with 1% tropicamide (Falcon Pharmaceuticals). Mice were anesthetized using a drug cocktail of ketamine (20 mg/ml) and xylazine (1.7 mg/ml), and mice were exposed to the 85,000-lux LED 100 W white light (IP65, ZHMA) for 1 min. The first cohort of mice was sacrificed immediately and eyes were enucleated and used as the white light exposed control(D+WL). The second cohort of mice was exposed to white light and dark readapted for 5 min prior to sacrifice and HPLC analysis(D+WL+D). The third cohort of mice was exposed to white light for 1 min and then exposed to green laser light (532 nm) for either 30 s or 120 s, followed by a dark adaptation for 5 min(D+WL+D+GL). Mice kept under dark without any light exposure as dark adapted control (D). Mice were then sacrificed, and their eyes were enucleated. The eyes collected from each cohort were subjected to lipid extraction and HPLC analysis for retinoid sans previously described (9).

### Long-term dark adaptation

To determine the photodegradation of ocular retinoids in mice both WT and *Stra6*^*−/−*^ on albino and pigmented backgrounds were used for this experiment. After weaning WT and *Stra6*^*−/−*^mice were raised in a dark room without exposure to any light for 4 weeks. For the control, mice were raised on a dark/light cycle for 4 weeks after weaning. After the experimental period, mice were sacrificed, and eyes were enucleated for retinoid analysis by HPLC.

### Electroretinogram

2 months old WT and *Stra6*^*−/−*^ mice in both albino and pigmented background were dark adapted overnight for retina function analysis by electroretinography. Overnight dark-adapted mice were anesthetized by rodent anesthetic cocktail (20 mg × ml−1 ketamine and 1.75 mg × ml−1 xylazine), and the pupils were dilated with 1% tropicamide (Bausch and Lomb). Throughout the experimental procedure each mouse was placed on a temperature-regulated heating pad. Diagnosys Celeris rodent ERG device (Diagnosys) with an Ag-AgCL cornea electrode was used for recordings. Scotopic responses were obtained in the dark with 10 steps of white light, flash stimulus, ranging from 0.001 to 20 cd s × m^−2^. The duration of the inter-stimulus intervals increased from 4 s for low-luminance flashes to 90 s for the highest stimuli. After 7 min of light adaptation, cone ERGs were recorded with strobe-flash stimuli (0.32–63 cd s × m^−2^) superimposed on the adapting field.

### Isolation of mouse rhodopsin

Both albino and pigmented WT and *Stra6*^*−/−*^ were dark-adapted overnight. Next day, eyes were collected from these mice under dim red light. Eyes were either stored at −80 °C or used immediately. Rhodopsin was purified as described previously (22). Briefly, eyes were homogenized gently with a glass–glass homogenizer in the buffer consisting of 20 mM bis-tris propane (pH 7.5), 150 mM NaCl, 1 mM EDTA, and protease inhibitor cocktail in the dark. The homogenates were centrifuged for 15 min at 16,000*g* in a benchtop Eppendorf centrifuge at 4 °C. Supernatants were discarded, and pellets were solubilized in 20 mM bis-tris-propane (pH 7.5), 100 mM NaCl, 20 mM n-dodecyl-β-D-maltoside, and protease inhibitor cocktail for 1 h at 4 °C on a rotating platform. The membrane lysates were centrifuged at 16 000*g* for 60 min at 4 °C. The resulting supernatants were mixed with 1D4-immunoaffinity resin (6 mg of 1D4 anti-rhodopsin antibody/ml resin) equilibrated with 20 mM bis-tris-propane (pH 7.5), 100 mM NaCl, and 2 mM n-dodecyl-β-D-maltoside and incubated for 1 h at 4 °C on a rotating platform. Then, the resin-supernatant mixture was transferred to the column, flow-through was collected and the resin was washed with 20 mM BTP (pH 7.5), 500 mM NaCl and 2 mM n-dodecyl-β-D-maltoside. Rhodopsin was eluted with 20 mM BTP (pH 7.5), 100 mM NaCl, and 2 mM n-dodecyl-β-D-maltoside supplemented by the addition of 1D4 peptide (TETSQVAPA) at a final concentration of 0.1 mg × ml^−1^. The UV-visible spectra of the eluted proteins were measured with a UV-visible Cary 60 spectrophotometer. The concentration of rhodopsin was calculated using the extinction coefficient ε_500_ = 40,600 M^−1^ cm^−1^ (48). The concentration of ligand-free opsin was calculated using the extinction coefficient ε_280_ = 81,200 M^−1^ cm^−1^.

### Isolation of bis-retinoids

Mouse eye cups (4–10 eyes/sample) were homogenized in Dulbecco's phosphate-buffered saline using a tissue grinder in the presence of chloroform/methanol (1:1). Extraction was performed according to a previously published protocol (49). Briefly, the sample was extracted three times with addition of chloroform and centrifuged at 1000*g* for 5 min. After passage through a reversed phase (C18 Sep-Pak; Millipore) cartridge with 0.1% trifluoroacetic acid in methanol, the extract was concentrated by evaporation of solvent under gas and was redissolved in 50% methanolic chloroform (up to 10 eyes/30 ml of solvent). An Alliance system (Waters Corp) equipped with 2695 Separation Module, 2996 Photodiode Array Detector, 2475 Multi λ Fluorescence Detector was used for HPLC analysis. For compound elution, an Atlantis dC18 (3 μm, 4.6 × 150 mm; Waters) reverse phase column was used for the stationary phase and for the mobile phase a gradient of acetonitrile in water with 0.1% trifluoroacetic acid, 75 to 90% acetonitrile (0–30 min), 90 to 100% acetonitrile (30–40 min). 100% acetonitrile (40–80 min) with a flow rate of 0.5 ml/min. Detection by photodiode array was set at 430 and 490 nm.

### Statistical analysis

Statistical analyses were performed using either unpaired two-tailed Student’s *t* test or one-way ANOVA using GraphPad Prism 8.0 software. An alpha level of *p* < 0.05 was considered significant. Data are expressed as mean values ± standard deviation.

## Data availability

The data that support the findings of this study are available from the corresponding author upon reasonable request.

## Supporting information

This article contains supporting information.

## Conflict of interest

The authors declare no conflicts of interest with the content of this article.
