# Supplementary material for: Unraveling the mystery of ocular retinoid turnover: Insights from albino mice and the role of STRA6
Source: J Biol Chem. 2024 Feb 21;300(3):105781. doi: 10.1016/j.jbc.2024.105781 (PMC10950888; doi:10.1016/j.jbc.2024.105781)
Supplement: Supporting Information [file mmc1.pdf]

Supporting Information

Unraveling the Mystery of Ocular Retinoid Turnover: Insights from Albino Mice and the Role of STRA6

Srinivasagan Ramkumar[1], Beata Jastrzebska[1], Diego Montenegro[2,3], Janet R. Sparrow[2,3], and Johannes von Lintig[1,*]

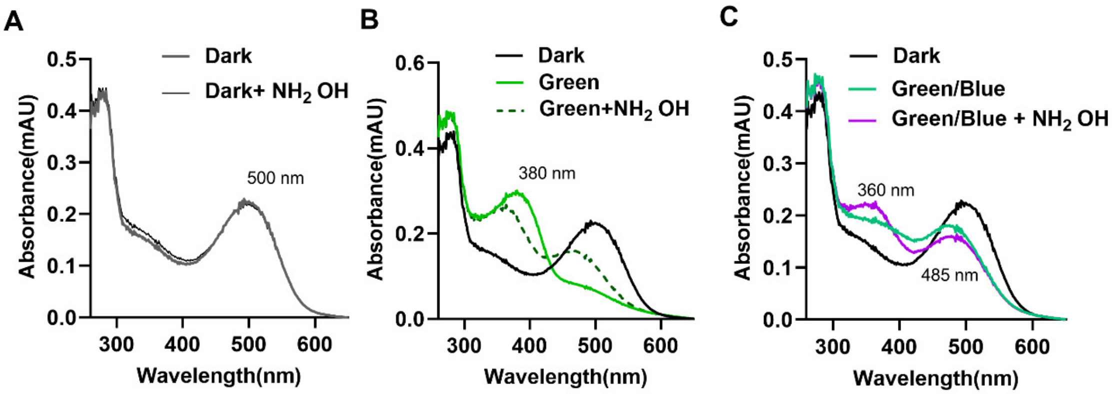

Figure S1. Hydroxylamine treated bovine rhodopsin. UV-visible spectra of bovine rhodopsin detergent-extracted from rod outer segments treated with hydroxylamine (A) Dark-adapted, (B) Green laser light exposed (C) Green and blue laser light exposed.

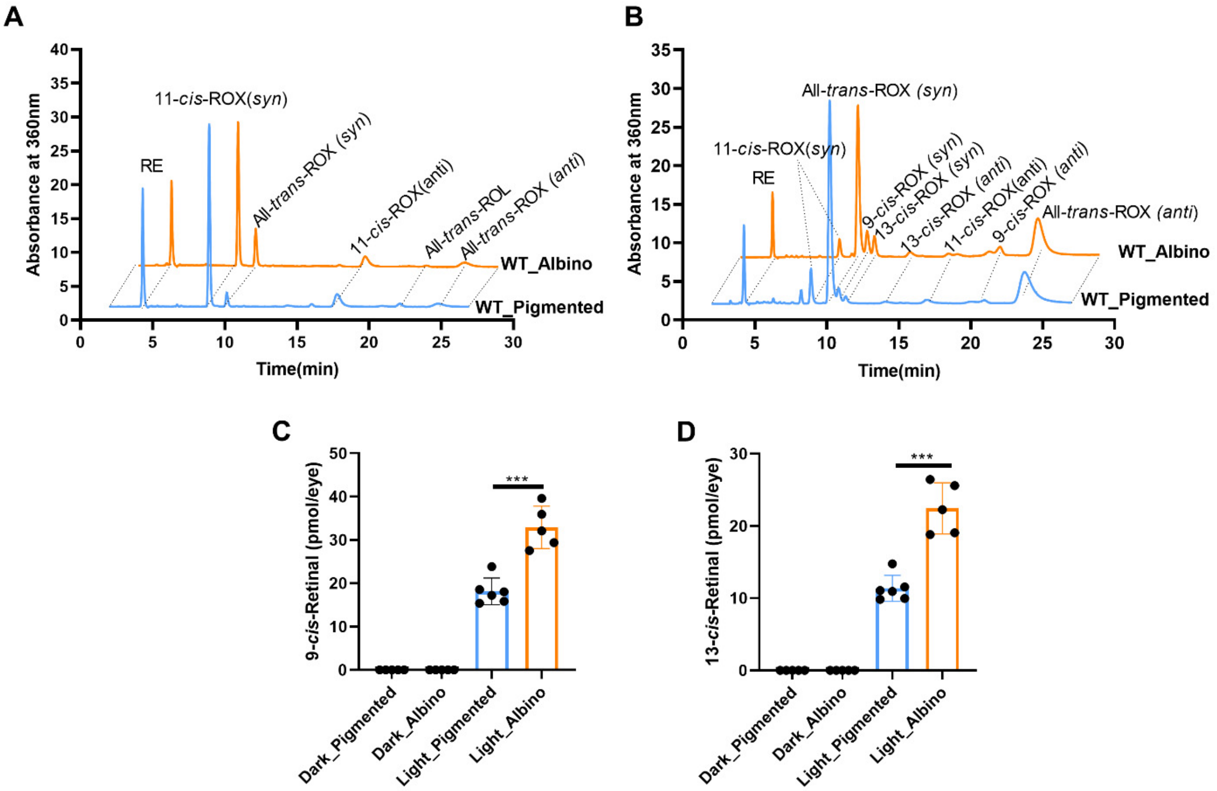

Figure S2. Ocular retinoid composition of dark adapted and bright white light exposed pigmented and albino WT mice. (A) HPLC trace at 360nm of ocular retinoid extract from dark adapted albino and pigmented WT mice. (B) HPLC trace at 360 nm of ocular retinoid extracts from dark-adapted albino and pigmented WT mice exposed to 85,000-lux LED bright white light for 1 min. The peaks for the individual retinal were marked. The retinal diastereomers were converted to corresponding retinal oximes (ROX) during ocular retinal extraction and these oximes exist as *syn* and *anti*-isomers. (C&D) The amount of 9-*cis*- and 13-*cis*-retinal, respectively from dark-adapted and dark adapted followed with 1min bright white light (85,000-lux) exposed albino and pigmented WT mice (n=5-6). Values are displayed as mean ± SD and statistical analyses were performed by

unpaired two-tailed Student's *t*-test ***$p < 0.0001$. POS, Photoreceptor Outer Segment; RPE, Retinal pigment epithelium; RE, Retinyl esters, ROX; Retinal oximes; ROL, Retinol.

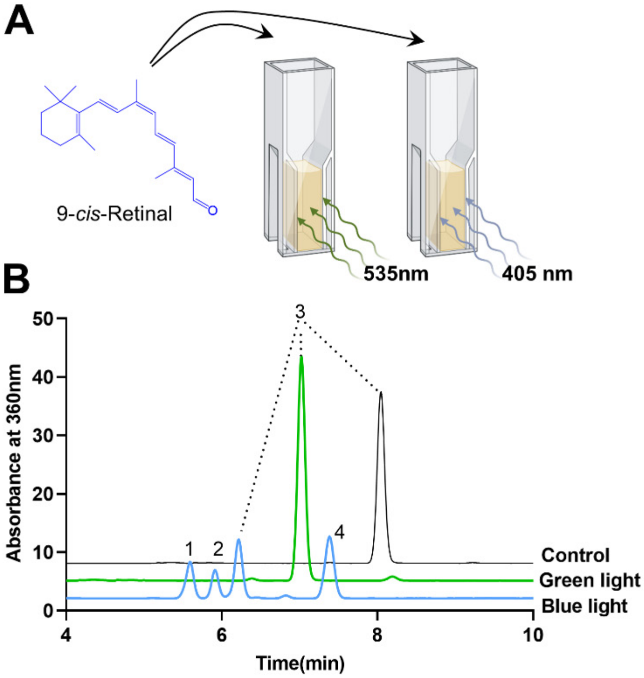

Figure S3. *In vitro* isomerization assay. (A) Scheme of *in vitro* isomerization of 9-*cis*-retinal by monochromatic green (535 nm) and blue (405 nm) laser light for 2 seconds. (B) HPLC trace for no light exposure control (black trace), green laser (green trace), and blue laser (blue trace) light exposure of *9-cis*-retinal. Peak 1, 11-*cis*-retinal, peak 2, 13-*cis*-retinal, peak 3, 9-*cis*-retinal, and peak 4, all-*trans*-retinal.

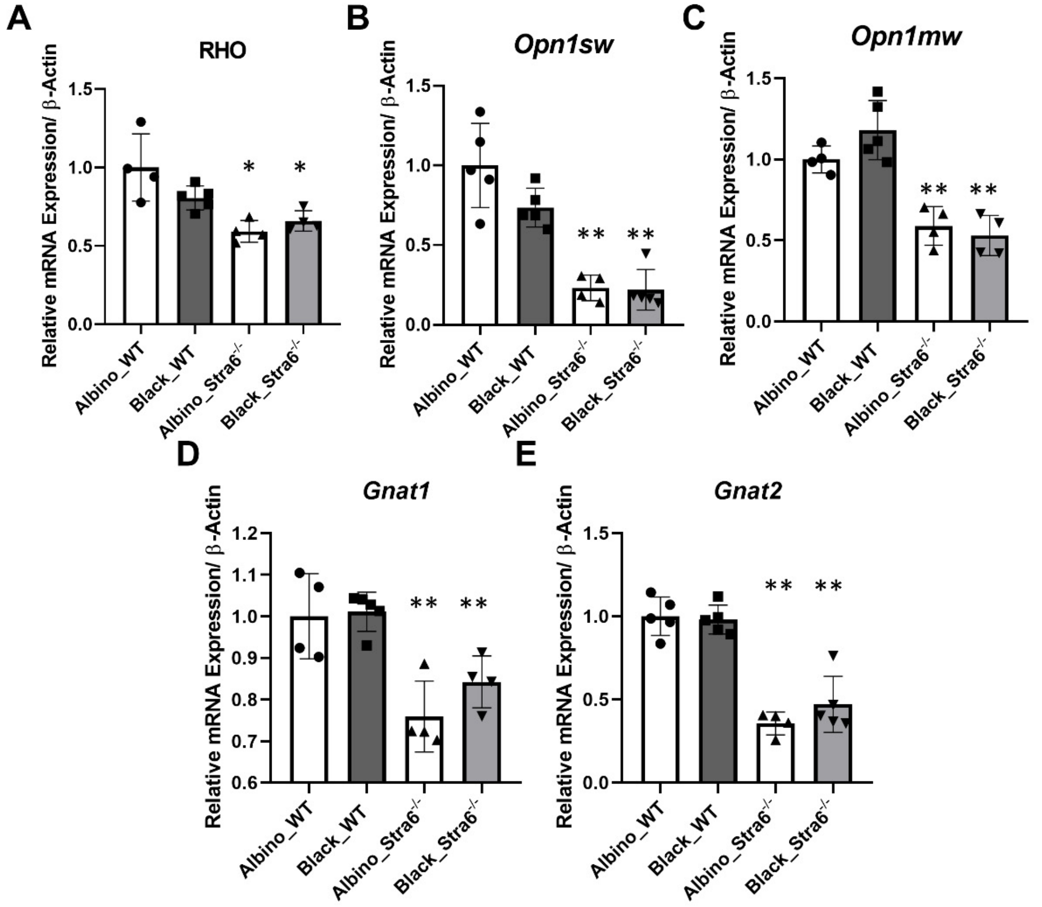

Figure S4. STRA6-deficient mice display reduced expression of cone and rod opsins. (A,B) Quantitative RT-PCR analysis for (A) *Rho*, (B), *Opn1sw*, (C) *Opn1mw*, (D) *Gnat1*, and (E) *Gnat2* in total RNA preparations of the retina in two-months-old albino and pigmented *Stra6*$^{-/-}$ mice and WT mice (n=4-5 per genotype). Statistical analyses were performed by comparing albino WT to pigmented and albino *Stra6*$^{-/-}$ mice, respectively, using unpaired two tail Student-*t* test. *, $p < 0.05$; **, $p < 0.005$.

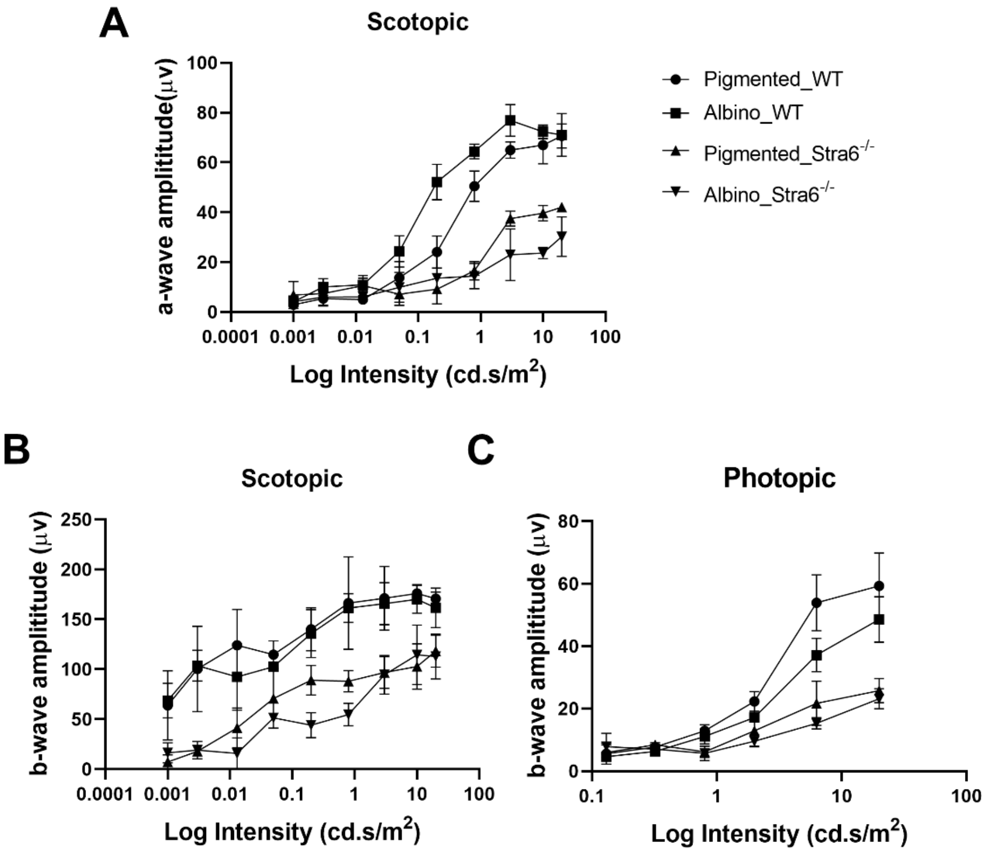

Figure S5. ERG responses of albino and pigmented STRA6-deficient and WT mice. ERG responses were recorded from two-month old *Stra6*$^{-/-}$ and age-matched WT mice (n=3 per age and genotype) (A, B) A and b-wave amplitudes under scotopic conditions (C) b-wave amplitude under photopic conditions.
